# Endophytic Fungi Associated with *Aquilaria sinensis* (Agarwood) from China Show Antagonism against Bacterial and Fungal Pathogens

**DOI:** 10.3390/jof8111197

**Published:** 2022-11-14

**Authors:** Tian-Ye Du, Samantha C. Karunarathna, Xian Zhang, Dong-Qin Dai, Ausana Mapook, Nakarin Suwannarach, Jian-Chu Xu, Steven L. Stephenson, Abdallah M. Elgorban, Salim Al-Rejaie, Saowaluck Tibpromma

**Affiliations:** 1 Center for Yunnan Plateau Biological Resources Protection and Utilization, College of Biological Resource and Food Engineering, Qujing Normal University, Qujing 655011, China; 2 Center of Excellence in Fungal Research, Mae Fah Luang University, Chiang Rai 57100, Thailand; 3 School of Science, Mae Fah Luang University, Chiang Rai 57100, Thailand; 4 Research Center of Microbial Diversity and Sustainable Utilization, Faculty of Science, Chiang Mai University, Chiang Mai 50200, Thailand; 5 Centre for Mountain Futures, Kunming Institute of Botany, Kunming 650201, China; 6 Department of Biological Sciences, University of Arkansas, Fayetteville, AR 72701, USA; 7 Department of Botany and Microbiology, College of Science, King Saud University, Riyadh 11451, Saudi Arabia; 8 Department of Pharmacology & Toxicology, College of Pharmacy, King Saud University, Riyadh 11451, Saudi Arabia

**Keywords:** agarwood, antibacterial activities, antifungal activities, antimicrobial activities, *Lasiodiplodia*

## Abstract

Agarwood is the most expensive non-construction wood product in the world. As a therapeutic agent, agarwood can cure some diseases, but few studies have been carried out on the antagonistic abilities of endophytic fungi associated with agarwood. Agarwood is mainly found in the genus *Aquiaria*. The objectives of this study are to understand the antimicrobial activities and their potential as biocontrol agents of the endophytic fungi of *Aquilaria sinensis*. First, fresh samples of *A. sinensis* were collected from Yunnan and Guangdong Provinces in 2020–2021, and the endophytic fungi were isolated and identified to genus level based on the phylogenetic analyses of the Internal Transcribed Spacer (ITS) region. In this bioassay, 47 endophytic strains were selected to check their bioactivities against three bacterial pathogens viz. *Erwinia amylovora, Pseudomonas syringae,* and *Salmonella enterica*; and three fungal pathogens viz. *Alternaria alternata, Botrytis cinerea,* and *Penicillium digitatum*. The antibiosis test was carried out by the dual culture assay (10 days), and among the 47 strains selected, 40 strains belong to 18 genera viz. *Alternaria*, *Annulohypoxylon*, *Aspergillus*, *Botryosphaeria*, *Colletotrichum*, *Corynespora*, *Curvularia*, *Daldinia*, *Diaporthe*, *Fusarium*, *Lasiodiplodia*, *Neofusicoccum*, *Neopestalotiopsis*, *Nigrospora*, *Paracamarosporium*, *Pseudopithomyces*, *Trichoderma*, *Trichosporon* and one strain belongs to Xylariaceae had antimicrobial activities. In particular, *Lasiodiplodia* sp. (YNA-D3) showed the inhibition of all the bacterial and fungal pathogens with a significant inhibition rate. In addition, the strains viz; *Curvularia* sp. (GDA-3A9), *Diaporthe* sp. (GDA-2A1), *Lasiodiplodia* sp. (YNA-D3), *Neofusicoccum* sp. (YNA-1C3), *Nigrospora* sp. (GDA-4C1), and *Trichoderma* sp. (YNA-1C1) showed significant antimicrobial activities and are considered worthy of further studies to identify individual fungal species and their bioactive compounds. This study enriches the diversity of endophytic fungi associated with agarwood, and their potential antagonistic effects against bacterial and fungal pathogens.

## 1. Introduction

*Aquilaria* Lam. (Thymelaeaceae Juss.) is the main genus that can produce agarwood [1,2]. Agarwood, a fragrant, dark, and resinous heartwood is the most expensive non-construction wood product in the world [3,4]. In China, agarwood is used in traditional Chinese medicine, and only *A*. *sinensis* (Lour.) Spreng. is the main agarwood tree species cultivated in Guangdong, Guangxi, Hainan, and Yunnan Provinces [4,5,6,7,8]. Current research on endophytic fungi associated with *A*. *sinensis* mainly focuses on the agarwood formation ability of the endophytic fungi [9,10], and only a few of the *A*. *sinensis* associated endophytic fungi have been studied for antimicrobial activities via dual culture assay [11]. In a previous study, 38 endophytic strains have been reported to have antimicrobial activities, for example, *Botryosphaeria rhodina* (Berk. & M.A. Curtis) Arx, *Cladosporium edgeworthiae* H. Zhang & Z.Y. Zhang, *Fusarium oxysporum* Schltdl., and *Guignardia mangiferae* A.J. Roy showed antimicrobial activities [12]; and a variety of important secondary metabolites with antibacterial and antimicrobial activities have been extracted from *Nemania aquilariae* Tibpromma & Zhang Lu [10]. However, the microorganisms that can be inhibited by agarwood are not clear enough, thus it is necessary to continue the research on the microbial spectrum of agarwood [13].

In this study, endophytic fungi associated with agarwood isolated from different plant tissues were used to test their antagonistic abilities against three pathogenic bacteria viz. *Erwinia amylovora* (Burrill) Winslow et al., *Pseudomonas syringae* van Hall, and *Salmonella* enterica (ex Kauffmann and Edwards) Le Minor and Popoff; and three pathogenic fungi viz. *Alternaria alternata* (Fr.) Keissl., *Botrytis cinerea* Pers., and *Penicillium digitatum* (Pers.) Sacc.

## 2. Materials and methods

### 2.1. Sample Collection and Isolation

#### 2.1.1. Sample Collection

Fresh samples of *A. sinensis* were collected three times; i.e., two times in Yunnan Province (21°55′48″ N, 101°15′36″ E, in November 2020; 22°21′09″ N, 101°01′06″ E, in September 2021) and one time in Guangdong Province (21°49′48″ N, 111°40′12″ E, in December 2020). Samples from Yunnan Province are denoted YNA, while from Guangdong Province are denoted GDA. The leaves and twigs of healthy plants, and the branches and twigs with agarwood dark resin were collected. Branch cutters, knives, and saws were used to cut the samples and they were cleaned with 75% alcohol before and after use. After collection, the fresh samples were placed in a thermal insulation ice box, brought back to the laboratory, and placed in the 4 °C refrigerators until the endophytic fungi are isolated.

#### 2.1.2. Isolation of Endophytic Fungi

Du et al. [14] with some adjustments was followed for the isolation of endophytic fungi in fresh agarwood samples. The bark of fresh samples was removed and then washed under running water, transferred to a laminar flow hood and the samples were cut into small pieces (0.5 cm × 0.5 cm) by sterilized knives and blades (sterilized with 75% alcohol). The surface disinfection steps of each sample are washed in sterile water, 75% alcohol for 30 s, 2.5% sodium hypochlorite for 1 min, and 75% alcohol for 30 s, finally, samples were washed in sterile water three times, and transferred to the sterilized filter paper to absorb the water. All tools were dipped in 95% alcohol and flamed before and after use. All the steps were done in a laminar flow hood. Five sterilized small pieces were placed in each 90 mm potato dextrose agar (PDA) plate (Ampicillin was added), and incubated at 28 °C for 14 days. During incubation, plates were checked every two days and the fresh mycelia were transferred to new 60 mm PDA plates to get pure cultures. The pure cultures were used for DNA extraction. Living pure cultures were deposited in the Zhongkai University of Agriculture and Engineering Culture Collection (ZHKUCC), China.

### 2.2. Endophytic Fungi Identification

#### 2.2.1. DNA Extraction, PCR Amplification and Sequencing

Ten days old fresh mycelia were used for DNA extraction using the Biospin Fungus Genomic DNA Extraction Kit–BSC14S1 (BioFlux, Hangzhou, China), following the manufacturer’s instructions [15]. Polymerase chain reaction (PCR) was used to amplify the ITS gene (internal transcribed spacer 1, 5.8S ribosomal RNA gene and internal transcribed spacer 2), using primers ITS5/ITS4 [16]. The PCR amplification was followed Du et al. [17], and the total volume of PCR mixtures for amplifications was 25 μL, with 94 °C: 3 min, (94 °C: 30 s, 55 °C: 50 s, 72 °C: 90 s) × 35 cycles, 72 °C: 10 min, final 4 °C. Finally, PCR products were purified and sequenced by Qinke Biotech Co., Kunming, China.

#### 2.2.2. Phylogenetic Analyses

Phylogenetic analyses are widely used in the identification of endophytic fungi, and the ITS gene is commonly used to primarily identify endophytic fungi to genus level [18,19,20,21,22]. In this study, to confirm the endophytic fungal genera, the ITS phylogenetic analyses were performed by Randomized Accelerated Maximum Likelihood (RAxML) analyses according to the parameters described in Dissanayake et al. [15]. The obtained sequences of the forward and reverse were merged in Geneious (9.1.2), and the merged sequences were subjected to BLAST (https://blast.ncbi.nlm.nih.gov/Blast.cgi?PAGE_TYPE=BlastSearch, accessed on 18 September 2022). Based on the BLAST search, the closest sequences were retrieved from the aNationl Center for Biotechnology Information (NCBI) (https://www.ncbi.nlm.nih.gov/, accessed on 18 September 2022). The sequences were aligned in the online website MAFFT v.7 (https://mafft.cbrc.jp/alignment/server/, accessed on 18 September 2022) [23], and automatic cutting was done in trimAl.v1.2rev59. BioEdit v. 7.0.5.2 [24] was used to manually combine the sequences, and subsequently, multiple sequence alignments were converted from FASTA to PHYLIP in ALTER (http://www.sing-group.org/ALTER/, accessed on 18 September 2022) [25]. The RAxML tree was run using the PHYLIP file, in RAxML-HPC BlackBox (8.2.12) [26,27] on the CIPRES Science Gateway platform (https://www.phylo.org/portal2/home.action, accessed on 18 September 2022) [28], with the GTR+I+G model of evolution. The final tree was visualized in FigTree v. 1.4.2 (http://tree.bio.ed.ac.uk/software/figtree/, accessed on 18 September 2022) [29], and edited in Microsoft PowerPoint 2010. The sequences generated in this study were uploaded to NCBI (https://submit.ncbi.nlm.nih.gov/subs/, accessed on 18 September 2022) to obtain the GenBank numbers (Table 1).

### 2.3. Pre Dual Culture Assay for Antibiosis Test (Pretest)

The ability of endophytic fungal isolates to inhibit the growth of pathogens was evaluated by the dual culture technique [30]. The six pathogens (three bacterial pathogens viz. *E. amylovora*, *P*. *syringae*, and *S*. *enterica*; and three fungal pathogens viz. *A*. *alternata*, *B*. *cinerea*, and *P*. *digitatum*) used in this study were obtained from the China General Microbiological Culture Collection Center (CGMCC).

The pretest is a screening test conducted before the formal test. We used 47 strains from all isolated strains for the pretest. The 47 endophytic fungi strains and six pathogenic strains (Table 2) were incubated at 28 °C for 10 days before the test. Fungi were cultivated on PDA, while bacteria were cultivated in nutrient agar (NA). After 10 days of incubation, the fungal colonies were cut into 0.4 cm diameter discs (sterilized plastic straw) in the laminar flow cabinet, then endophytic fungi and pathogenic fungi were inoculated in the same 90 mm PDA plates, and endophytic fungi and pathogenic bacteria (bacteria scraped with 0.4 cm wide strip) were inoculated in the same 90 mm NA plates. The control was inoculated with only pathogens. All the plates were incubated at 28 °C for 10 days. After 10 days, the colony growth of the test group and the control group were checked and recorded. According to the test results, we compared the colony diameters of the test group and the control group, and then endophytic fungi that can inhibit three pathogenic fungi or three pathogenic bacteria were selected. These selected strains with antagonistic activities were used for formal testing.

### 2.4. Dual Culture Assay for Antibiosis Test (Formal Test)

#### 2.4.1. Methods of Dual Culture Assay

The test method is similar to the pretest. According to the results of the pretest, among 47 endophytic fungi, 25 strains were able to inhibit pathogenic bacteria, 40 strains were able to inhibit pathogenic fungi, and 18 strains were able to inhibit both pathogenic fungi and bacteria. Therefore, 47 endophytes and six pathogens were incubated at 28 °C for 10 days before the formal test. Fungi were incubated on PDA, while bacteria were incubated in NA.

The endophytic and pathogenic fungi grown on PDA plates were cut into small fungal discs (0.4 cm diam.) using a sterilized plastic straw in laminar. Then, the 25 selected endophytic fungal strains were inoculated with three pathogenic bacteria (0.4 cm wide strip) in the same NA plates, and each test was replicated three times (total of 25 × 3 × 3 = 225 plates). The 40 selected endophytic strains were inoculated with three pathogenic fungi in the same PDA plates, and each test was replicated three times (total of 40 × 3 × 3 = 360 plates). The pathogens were inoculated on the left of the petri dish, while the endophytic fungi were inoculated on the right by keeping a space of 6 cm between the pathogens and endophytes. Negative controls were set in the antibiosis tests of each pathogen. The control group used the same culture medium as the test group. The pathogen was inoculated on the left of the medium, while nothing was inoculated on the right. Controls were incubated under the same conditions as the test groups. After inoculation, petri dishes were incubated at 28 °C for 10 days. While incubating, they were observed, photographed and the diameter of the pathogens in the test group and the control group was measured every two days.

#### 2.4.2. Calculation and Analysis of Inhibition Rate

According to the test results, the data were processed and analyzed. The antibiosis effects and the degree of endophytic fungi effect on pathogens can be expressed by calculating the inhibition rate of endophytic fungi on the growth diameter of pathogens. The inhibition rate was calculated according to the method described in Gao et al. [58] and Rajani et al. [59], and the calculation formula used is as follows:

Inhibition% = (Cd − Td)/(Cd − 0.4) × 100(1)

Notes: Cd = radial growth of the pathogen in pure control culture, Td = radial growth of the pathogen in dual culture. The width of the original fungal discs and bacterial strip in this test is 0.4 cm.

#### 2.4.3. Statistical Analyses

The statistical analyses of the inhibition rate were carried out in Microsoft Excel 2010. The measured data (colony diameter) were recorded in an excel table. The inhibition rate and average inhibition rate were obtained by the formula. The standard deviation (SD) reflects the dispersion degree of a data set, and the values were obtained by inserting the function (STDEV) of standard deviation into the excel table. In addition, clustered column graphs were inserted in the excel table based on the average inhibition rate and edited in Microsoft Excel 2010.

## 3. Results

### 3.1. Results of Sample Collection and Isolation

In this study, agarwood samples were collected from Guangdong and Yunnan Provinces. The fresh samples were isolated to obtain pure cultures for molecular analyses and antibiosis tests. Figure S1 shows the culture morphologies of 47 endophytic fungi strains, and in Table 1, we list the host, collection site, and other information of 47 endophytic fungi used in this study.

### 3.2. Single Gene Phylogenetic Analyses

The single-gene phylogenetic analyses were carried out by constructing an RAxML phylogenetic tree based on ITS. The RAxML analyses gave a final ML optimization likelihood value of −12,190.561600. The matrix had 567 distinct alignment patterns, with 19.72% of undetermined characters or gaps. Parameters for the GTR+I+G model of the ITS were as follows: estimated base frequencies A = 0.249972, C = 0.260278, G = 0.245555, T = 0.244194; substitution rates AC = 1.190651, AG = 3.363586, AT = 2.316682, CG = 1.153165, CT = 3.693909, GT = 1.000000; proportion of invariable sites I = 0.105968; and gamma distribution shape parameter α = 0.446892.

**Figure 1:**
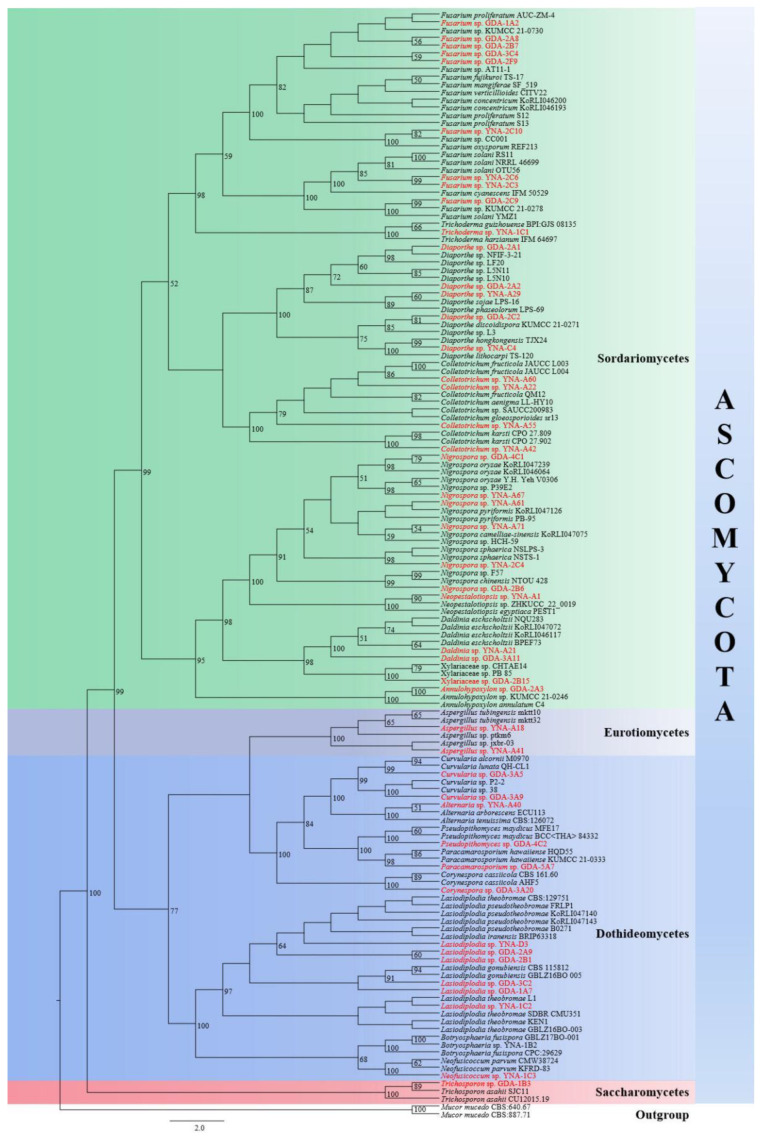
A RAxML single gene phylogenetic tree of 47 endophytic fungi strains and their related sequences based on ITS. Bootstrap support values for maximum likelihood (ML) equal to or higher than 50% are indicated above the branches. The endophytic fungi with original strain numbers isolated in this study are marked with red font.

The final RAxML tree is shown in Figure 1. The 47 strains are distributed in four classes in Ascomycota, viz. Dothideomycetes, Eurotiomycetes, Saccharomycetes, and Sordariomycetes. According to the BLAST results and phylogenetic analyses, 46 strains were identified at the genus level, and they belong to 18 genera. While one of our strains (GDA-2B15) is closest to two strains of Xylariaceae viz. (CHTAE14) and (PB-85), therefore, GDA-2B15 was identified as a member of Xylariaceae in this paper.

The results can be summarized as 47 endophytic fungi strains belong to Ascomycota Caval.-Sm., of which 30 strains belong to Sordariomycetes O.E. Erikss. & Winka (63.83%), 14 strains belong to Dothideomycetes O.E. Erikss. & Winka (29.79%), two strains belong to Eurotiomycetes O.E. Erikss. & Winka (4.26%), and one strain belongs to Saccharomycetes G. Winter (2.13%).

### 3.3. Dual Culture Assay for Antibiosis Test (Pretest)

A total of 47 endophytic fungi strains were tested on six pathogens under the same conditions. The results showed that 18 strains had inhibitory effects on all six pathogens, seven strains had inhibitory effects on all three pathogenic bacteria, and 22 strains had inhibitory effects on all three pathogenic fungi. Therefore, 25 strains had inhibitory effects on all three pathogenic bacteria, and 40 strains had inhibitory effects on all three pathogenic fungi. Therefore, 25 strains and 40 strains were used to conduct formal tests on three pathogenic bacteria and three pathogenic fungi respectively.

### 3.4. Dual Culture Assay for Antibiosis Test (Formal Test)

**Figure 2:**
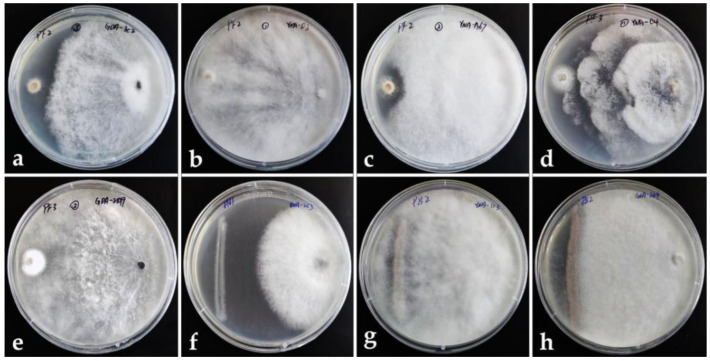
Dual culture assay. Left: pathogen. Right: endophytic fungus. (**a**–**c**) Endophytic fungi dominate against the pathogenic fungus PF2. (**d**,**e**) Endophytic fungi dominate against pathogenic fungus PF3. (**f**) Endophytic fungi dominate against pathogenic bacterium PB1. (**g**,**h**) Endophytic fungi dominate against pathogenic bacterium PB2.

Through the results of the pretest, we carried out the formal test with the selected strains (25 endophytic fungi for pathogenic bacteria, and 40 endophytic fungi for pathogenic fungi) By calculating the inhibition rate through the formula, the strains whose inhibition rate was more than 60% were considered to have an inhibition effect, and the results recorded in Table 3 and only *Lasiodiplodia* sp.(YNA-D3) can inhibit all six pathogens, and its inhibition rate to pathogenic fungi is higher than bacteria pathogens (Inhibition rate: 93.30% to PF2-*B. cinerea*, 76.73% to PF3-*P. digitatum*, 75.90% to PF1-*A. alternata*, 74.07% to PB2-*P. syringae*, 63.33% to PB3-*S. enterica*, 63.64% to PB1-*E. amylovora*). Figure 2 shows the pictures of several endophytic fungi with significant inhibition rates to pathogens in the dual culture assay.

#### 3.4.1. Inhibition of 25 Endophytic Fungi on Three Pathogenic Bacteria

**Figure 3:**
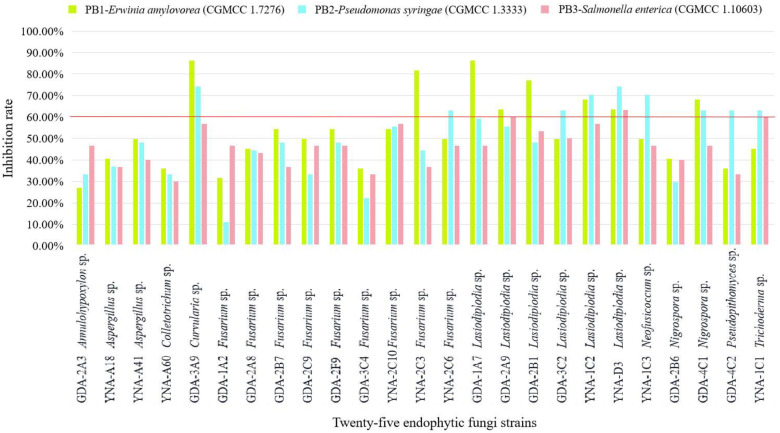
Inhibition rate of 25 endophytic fungi to three pathogenic bacteria. The inhibition ≥60% is considered a good inhibition effect.

The inhibitory effect (≥60%) of 25 endophytic fungi on pathogenic bacteria is shown in Figure 3 and Table 3, and the inhibitory effect is ranked as *E. amylovora* (CGMCC 1.7276) > *P. syringae* (CGMCC 1.3333) > *S. enterica* (CGMCC 1.10603). For *S. enterica* (CGMCC 1.10603), there is almost no inhibitory effect.

For PB1-*E. amylovora* (CGMCC 1.7276), eight strains showed inhibitory effects (Table 3 and Figure 3), and the three strains with the highest inhibition rate are *Curvularia* sp. (GDA-3A9, 86.36%), *Lasiodiplodia* sp. (GDA-1A7, 86.36%), and *Fusarium* sp. (YNA-2C3, 81.82%). Among the eight strains, the genus *Lasiodiplodia* Ellis & Everh. has the highest number of strains (five strains).

For PB2-*P. syringae* (CGMCC 1.3333), nine strains showed inhibitory effects (Table 3 and Figure 3), and the three strains with the highest inhibition rate are *Curvularia* sp. (GDA-3A9, 74.07%), *Lasiodiplodia* sp. (YNA-D3, 74.07%), and *Lasiodiplodia* sp. (YNA-1C2, 70.37%). Among the nine strains, the genus *Lasiodiplodia* has the largest number of strains (three strains).

For PB3-*S. enterica* (CGMCC 1.10603), three strains showed inhibitory effects (Table 3 and Figure 3), and *Lasiodiplodia* sp. (YNA-D3, 63.33%) had the strongest inhibitory effect, followed by *Lasiodiplodia* sp. (GDA-2A9, 60.00%), and *Trichoderma* sp. (YNA-1C1, 60.00%). Among the three strains, the genus *Lasiodiplodia* has the largest number of strains (two strains).

#### 3.4.2. Inhibition of 40 Endophytic Fungi on Three Pathogenic Fungi

**Figure 4:**
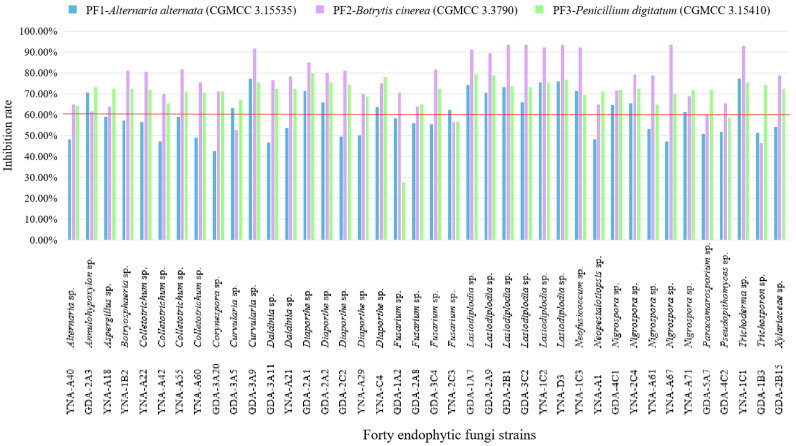
Inhibition rate of 40 endophytic fungi to three pathogenic fungi. The inhibition ≥60% is considered a good inhibition effect.

The inhibitory effect (≥60%) of 40 endophytic fungi on pathogenic fungi shows some good results in Figure 4 and Table 3, and the inhibitory effect is ranked as *B. cinerea* (CGMCC 3.3790) > *P. digitatum* (CGMCC 3.15410) > *A. alternata* (CGMCC 3.15535).

For PF1-*A. alternata* (CGMCC 3.15535), 18 strains showed inhibitory effects (Table 3 and Figure 4), among them, the three strains with the highest inhibition rate are *Curvularia* sp. (GDA-3A9, 77.07%), *Trichoderma* sp. (YNA-1C1, 77.07%), and *Lasiodiplodia* sp. (YNA-D3, 75.90%). Among the 18 strains, the genus *Lasiodiplodia* has the largest number of strains (six strains).

For PF2-*B. cinerea* (CGMCC 3.3790), 36 strains showed inhibitory effects (Table 3 and Figure 4), among them, the three strains with the highest inhibition rate are *Lasiodiplodia* sp. (GDA-3C2, 93.30%), *Lasiodiplodia* sp. (YNA-D3, 93.30%), and *Lasiodiplodia* sp. (GDA-2B1, 93.30%). Among the 36 strains, the genus *Lasiodiplodia* has the largest number of strains (six strains).

For PF3-*P. digitatum* (CGMCC 3.15410), 38 strains showed inhibitory effects (Table 3 and Figure 4), among them, the three strains with the highest inhibition rate are *Diaporthe* sp. (GDA-2A1, 79.87%), *Lasiodiplodia* sp. (GDA-1A7, 79.25%), and *Lasiodiplodia* sp. (GDA-2A9, 78.62%). Among the 38 strains, the genus *Lasiodiplodia* has the largest number of strains (six strains).

To sum up, the endophytic fungi used in this test have a good inhibitory effect on PF2-*B. cinerea* (CGMCC 3.3790), which can reach a 93.30% inhibition rate, however, for PB3-*S. enterica* (CGMCC 1.10603), there was almost no inhibitory effect, and the highest inhibitory rate was 63.33%. Among the inhibition results of endophytic fungi on these six pathogens, it can be seen that most fungi with inhibitory effect belong to the genus *Lasiodiplodia*, and *Lasiodiplodia* sp. (YNA-D3) showed the best inhibition effect on pathogens (anti-PB1 63.47%, anti-PB2 74.07%, anti-PB3 63.33%, anti-PF1 75.90%, anti-PF2 93.30%, and anti-PF3 76.73%).

## 4. Discussion

The 47 endophytic fungal strains isolated from agarwood were tested against six bacterial and fungal pathogens. The reasons for selecting these six pathogens are: few studies have been carried out on the pathogens of *A. sinensis* trees, thus no pathogenic strains of *A. sinensis* are available to be used, and these six pathogens can cause severe damages, their hosts and distribution are very wide and common [42,48,53,55].

The results of the dual culture assay showed that 40 endophytic fungi strains with antimicrobial activities out of 47 strains belong to 18 genera viz. *Alternaria* Nees, *Annulohypoxylon* Y.M. Ju, J.D. Rogers & H.M. Hsieh, *Aspergillus* P. Micheli ex Haller, *Botryosphaeria* Ces. & De Not., *Colletotrichum* Corda, *Corynespora* Güssow, *Curvularia* Boedijn, *Daldinia* Ces. & De Not., *Diaporthe* Nitschke, *Fusarium* Link, *Lasiodiplodia*, *Neofusicoccum* Crous, Slippers & A.J.L. Phillips, *Neopestalotiopsis* Maharachch., K.D. Hyde & Crous, *Nigrospora* Zimm., *Paracamarosporium* Wijayaw. & K.D. Hyde, *Pseudopithomyces* Ariyaw. & K.D. Hyde, *Trichoderma* Pers., and *Trichosporon* Behrend while one strain was identified as Xylariaceae Tul. & C. Tul., while their inhibitory effects on different pathogens were identified as different (Table 3). Among them, the strains of six genera (*Curvularia*, *Diaporthe*, *Lasiodiplodia*, *Neofusicoccum*, *Nigrospora*, and *Trichoderma*) showed relatively significant inhibition effects (Table 3) and the most significant of which is *Lasiodiplodia* sp. (YNA-D3), which can inhibit all six pathogens.

In previous studies, some agarwood endophytic fungal strains have been shown to have antimicrobial properties that are consistent with our results viz. *Botryosphaeria rhodina* [12], *Colletotrichum* sp. [12], *Diaporthe* sp. [60], *Fusarium equiseti* (Corda) Sacc. [61], *F. oxysporum* [12,61], *F. solani* (Mart.) Sacc. [61], *F. verticillioides* (Sacc.) Nirenberg [62], *Lasiodiplodia theobromae* (Pat.) Griffon & Maubl. [61], and *Xylaria mali* Fromme [63].

In addition, in this study, this is the first time that 13 genera of agarwood endophytic fungi are reported for antimicrobial activities viz. *Alternaria*, *Annulohypoxylon*, *Aspergillus*, *Corynespora*, *Curvularia, Daldinia*, *Neofusicoccum*, *Neopestalotiopsis*, *Nigrospora*, *Paracamarosporium*, *Pseudopithomyces*, *Trichoderma*, and *Trichosporon*. At the same time, nine genera viz. *Alternaria*, *Annulohypoxylon*, *Corynespora*, *Daldinia*, *Neofusicoccum*, *Neopestalotiopsis*, *Paracamarosporium*, *Pseudopithomyces*, and *Trichosporon* were reported as endophytic fungi of agarwood for the first time.

In this study, some potential fungal strains that can be used as biocontrol agents were screened (Table 3). *Botrytis cinerea* (CGMCC 3.3790) is one of the most destructive pathogens with a large number of hosts [53]. This pathogen is resistant to commonly used synthetic fungicides, so it is necessary to carry out more research on biological control strategies [53,54]. In this study, strains of the five genera viz. *Curvularia* sp., *Lasiodiplodia* sp., *Neofusicoccum* sp., *Nigrospora* sp., and *Trichoderma* sp. with inhibition rates to *B. cinerea* (CGMCC 3.3790) more than 90% were identified. These strains have the potential to be developed into fungicides against *B. cinerea* (CGMCC 3.3790).

In conclusion, this study enriches the diversity of the endophytic fungi of agarwood and their antagonistic potential against bacterial and fungal pathogens. The most significant fungal strain is *Lasiodiplodia* YNA-D3 which can inhibit all pathogens and needs further studies to identify and analyze its secondary metabolites with antimicrobial effects. In addition, in-depth studies on the endophytic fungi associated with agarwood are needed to develop effective biocontrol agents.

## Figures and Tables

**Table 1:** Original code, strain name, strain number, ITS GenBank accession number, class in Ascomycota, plant tissue, collection site, and date of collection of 47 fungal strains associated with *Aquilaria sinensis* used in this study. The contents in the table are arranged according to the genus of endophytic fungi.

Original Code	Strain Name	Strain Number	ITS GenBank Accession Number	Plant Tissue	Collection Site	Collection Date
**Dothideomycetes**						
YNA-A40	*Alternaria* sp.	ZHKUCC 22-0248	OP445267	Health leaves	Yunnan	November 2020
YNA-1B2	*Botryosphaeria* sp.	ZHKUCC 22-0249	OP450949	Agarwood resins	Yunnan	September 2021
GDA-3A20	*Corynespora* sp.	KUMCC 21-0302	OL455852	Agarwood resins	Guangdong	December 2020
GDA-3A5	*Curvularia* sp.	KUMCC 21-0287	OL455828	Agarwood resins	Guangdong	December 2020
GDA-3A9	*Curvularia* sp.	KUMCC 21-0291	OL455842	Agarwood resins	Guangdong	December 2020
GDA-1A7	*Lasiodiplodia* sp.	KUMCC 21-0224	OL548888	Agarwood resins	Guangdong	December 2020
GDA-2A9	*Lasiodiplodia* sp.	KUMCC 21-0252	OL455795	Agarwood resins	Guangdong	December 2020
GDA-2B1	*Lasiodiplodia* sp.	KUMCC 21-0254	OL455797	Agarwood resins	Guangdong	December 2020
GDA-3C2	*Lasiodiplodia* sp.	KUMCC 21-0324	OL548897	Agarwood resins	Guangdong	December 2020
YNA-1C2	*Lasiodiplodia* sp.	ZHKUCC 22-0251	OP450951	Agarwood resins	Yunnan	September 2021
YNA-D3	*Lasiodiplodia* sp.	ZHKUCC 22-0270	OP445276	Health branches	Yunnan	November 2020
YNA-1C3	*Neofusicoccum* sp.	ZHKUCC 22-0252	OP450952	Agarwood resins	Yunnan	September 2021
GDA-5A7	*Paracamarosporium* sp.	ZHKUCC 22-0247	OP439521	Health branches	Guangdong	December 2020
GDA-4C2	*Pseudopithomyces* sp.	ZHKUCC 22-0246	OP439520	Health branches	Guangdong	December 2020
**Eurotiomycetes**						
YNA-A18	*Aspergillus* sp.	ZHKUCC 22-0258	OP445263	Health leaves	Yunnan	November 2020
YNA-A41	*Aspergillus* sp.	ZHKUCC 22-0262	OP445268	Health leaves	Yunnan	November 2020
**Saccharomycetes**						
GDA-1B3	*Trichosporon* sp.	KUMCC 21-0230	OL455772	Agarwood resins	Guangdong	December 2020
**Sordariomycetes**						
GDA-2A3	*Annulohypoxylon* sp.	KUMCC 21-0246	OL455788	Agarwood resins	Guangdong	December 2020
YNA-A22	*Colletotrichum* sp.	ZHKUCC 22-0260	OP445265	Health leaves	Yunnan	November 2020
YNA-A42	*Colletotrichum* sp.	ZHKUCC 22-0263	OP445269	Health leaves	Yunnan	November 2020
YNA-A55	*Colletotrichum* sp.	ZHKUCC 22-0264	OP445270	Health leaves	Yunnan	November 2020
YNA-A60	*Colletotrichum* sp.	ZHKUCC 22-0265	OP445271	Health leaves	Yunnan	November 2020
GDA-3A11	*Daldinia* sp.	KUMCC 21-0293	OL455844	Agarwood resins	Guangdong	December 2020
YNA-A21	*Daldinia* sp.	ZHKUCC 22-0259	OP445264	Health leaves	Yunnan	November 2020
GDA-2A1	*Diaporthe* sp.	KUMCC 21-0244	OL455786	Agarwood resins	Guangdong	December 2020
GDA-2A2	*Diaporthe* sp.	KUMCC 21-0245	OL455787	Agarwood resins	Guangdong	December 2020
GDA-2C2	*Diaporthe* sp.	KUMCC 21-0271	OL455832	Agarwood resins	Guangdong	December 2020
YNA-A29	*Diaporthe* sp.	ZHKUCC 22-0261	OP445266	Health leaves	Yunnan	November 2020
YNA-C4	*Diaporthe* sp.	ZHKUCC 22-0269	OP445275	Health leaves	Yunnan	November 2020
GDA-1A2	*Fusarium* sp.	KUMCC 21-0219	OL548884	Agarwood resins	Guangdong	December 2020
GDA-2A8	*Fusarium* sp.	KUMCC 21-0251	OL455794	Agarwood resins	Guangdong	December 2020
GDA-2B7	*Fusarium* sp.	KUMCC 21-0260	OL455811	Agarwood resins	Guangdong	December 2020
GDA-2C9	*Fusarium* sp.	KUMCC 21-0278	OL455819	Agarwood resins	Guangdong	December 2020
GDA-2F9	*Fusarium* sp.	ZHKUCC 22-0244	OP439518	Agarwood resins	Guangdong	December 2020
GDA-3C4	*Fusarium* sp.	KUMCC 21-0326	OL548899	Agarwood resins	Guangdong	December 2020
YNA-2C10	*Fusarium* sp.	ZHKUCC 22-0253	OP450965	Agarwood resins	Yunnan	September 2021
YNA-2C3	*Fusarium* sp.	ZHKUCC 22-0254	OP450953	Agarwood resins	Yunnan	September 2021
YNA-2C6	*Fusarium* sp.	ZHKUCC 22-0256	OP450955	Agarwood resins	Yunnan	September 2021
YNA-A1	*Neopestalotiopsis* sp.	ZHKUCC 22-0257	OP445262	Health leaves	Yunnan	November 2020
GDA-2B6	*Nigrospora* sp.	KUMCC 21-0259	OL455810	Agarwood resins	Guangdong	December 2020
GDA-4C1	*Nigrospora* sp.	ZHKUCC 22-0245	OP439519	Health branches	Guangdong	December 2020
YNA-2C4	*Nigrospora* sp.	ZHKUCC 22-0255	OP450954	Agarwood resins	Yunnan	September 2021
YNA-A61	*Nigrospora* sp.	ZHKUCC 22-0266	OP445272	Health leaves	Yunnan	November 2020
YNA-A67	*Nigrospora* sp.	ZHKUCC 22-0267	OP445270	Health leaves	Yunnan	November 2020
YNA-A71	*Nigrospora* sp.	ZHKUCC 22-0268	OP445274	Health leaves	Yunnan	November 2020
YNA-1C1	*Trichoderma* sp.	ZHKUCC 22-0250	OP450949	Agarwood resins	Yunnan	September 2021
GDA-2B15	*Xylariaceae* sp.	KUMCC 21-0268	OL455829	Agarwood resins	Guangdong	December 2020

**Table 2:** Six pathogens were purchased from China General Microbiological Culture Collection Center (CGMCC). The PB (pathogenic bacteria) and PF (pathogenic fungi) are new codes created in this study to distinguish pathogenic bacteria and pathogenic fungi.

Pathogen	New Code	Strain Name	Strain Number	Effects of Pathogens	References
Pathogenic bacteria	PB1	*Erwinia amylovora*	CGMCC 1.7276	*Erwinia amylovora* causes a destructive plant disease that endangers many host species of Rosaceae Juss. (e.g., apple, blackberry, cotoneaster, pear, pyracantha, and raspberry)	[31,32,33,34,35,36,37,38]
	PB2	*Pseudomonas syringae*	CGMCC 1.3333	*Pseudomonas syringae* mainly harms plant hosts, including fruit trees (such as apples, hazelnuts and plums) and some field crops (such as beets, cabbage, cucumbers, oats, peas, rice, tobacco, and tomatoes), which cause major economic losses	[39,40,41]
	PB3	*Salmonella enterica*	CGMCC 1.10603	*Salmonella enterica* is a zoonotic pathogenic bacterium. It can cause acute gastroenteritis, and it causes other symptoms such as septicaemia, fever and/or abortion. The resistance of this pathogen to multiple antibiotics is a public threat to most Asian countries	[42,43,44,45,46]
Pathogenic fungi	PF1	*Alternaria alternata*	CGMCC 3.15535	*Alternaria alternata* is a pathogenic fungus, that infects important cash crops and lead to human and animal diseases. In the field of human diseases, *A. alternata* is considered to be one of the most important fungal allergens in the world, which are related to severe asthma and respiratory status	[47,48,49,50,51,52]
	PF2	*Botrytis cinerea*	CGMCC 3.3790	*Botrytis cinerea* is one of the most destructive pathogens, especially for food and fruits obtained in the field and storage room. Because the pathogen is resistant to commonly used synthetic fungicides, a number of research activities have been carried out, focusing on the development of biological control strategies for the pathogen	[53,54]
	PF3	*Penicillium digitatum*	CGMCC 3.15410	*Penicillium digitatum* is a main pathogenic fungus of postharvest decay of fruits belonging to Rutaceae Juss. This high host specificity leads to the loss of citrus fruits	[55,56,57]

**Table 3:** The results and inhibition rate percentage ± standard deviation of dual culture assay-formal test (10 days). “−” indicates that the dual culture assay-formal test of endophytic fungi against the pathogen has not been carried out. Taxa are arranged according to the alphabetical order of generic names.

Original Code	Strain Name	PB1-*Erwinia amylovora* (CGMCC 1.7276)	PB2-*Pseudomonas syringae* (CGMCC 1.3333)	PB3-*Salmonella enterica* (CGMCC 1.10603)	PF1-*Alternaria alternata* (CGMCC 3.15535)	PF2-*Botrytis cinerea* (CGMCC 3.3790)	PF3-*Penicillium digitatum* (CGMCC 3.15410)
YNA-A40	*Alternaria* sp.	–	–	–	48.27 ± 2.76	64.82 ± 0.07	64.15 ± 1.14
GDA-2A3	*Annulohypoxylon* sp.	26.94 ± 1.67	33.33 ± 0.00	46.67 ± 0.22	70.61 ± 0.03	61.47 ± 1.74	72.96 ± 0.58
YNA-A18	*Aspergillus* sp.	40.64 ± 0.42	37.04 ± 0.27	36.67 ± 1.56	58.85 ± 0.53	63.71 ± 1.24	72.33 ± 0.03
YNA-A41	*Aspergillus* sp.	49.77 ± 0.42	48.15 ± 1.10	40.00 ± 0.00	–	–	–
YNA-1B2	*Botryosphaeria* sp.	–	–	–	57.08 ± 0.63	81.02 ± 0.19	72.33 ± 0.03
YNA-A22	*Colletotrichum* sp.	–	–	–	56.50 ± 2.02	80.46 ± 0.04	71.70 ± 0.02
YNA-A42	*Colletotrichum* sp.	–	–	–	47.09 ± 2.63	69.85 ± 0.90	65.41 ± 1.10
YNA-A55	*Colletotrichum* sp.	–	–	–	58.85 ± 1.25	81.57 ± 0.00	71.07 ± 0.81
YNA-A60	*Colletotrichum* sp.	36.07 ± 2.92	33.33 ± 0.00	30.00 ± 0.67	48.85 ± 1.18	75.43 ± 0.31	70.44 ± 0.72
GDA-3A20	*Corynespora* sp.	–	–	–	42.39 ± 2.95	70.97 ± 0.10	71.07 ± 0.01
GDA-3A5	*Curvularia* sp.	–	–	–	62.96 ± 0.44	52.54 ± 2.18	67.30 ± 0.13
GDA-3A9	*Curvularia* sp.	86.30 ± 0.00	74.07 ± 0.27	56.67 ± 0.22	77.07 ± 0.02	91.62 ± 0.02	75.47 ± 0.17
GDA-3A11	*Daldinia* sp.	–	–	–	46.50 ± 0.17	76.55 ± 0.00	72.33 ± 0.13
YNA-A21	*Daldinia* sp.	–	–	–	53.56 ± 0.44	78.22 ± 0.07	72.33 ± 0.03
GDA-2A1	*Diaporthe* sp.	–	–	–	71.19 ± 0.01	84.92 ± 0.13	79.87 ± 0.01
GDA-2A2	*Diaporthe* sp.	–	–	–	65.90 ± 0.03	79.90 ± 0.24	75.47 ± 0.07
GDA-2C2	*Diaporthe* sp.	–	–	–	49.44 ± 2.08	81.02 ± 0.01	74.21 ± 0.01
YNA-A29	*Diaporthe* sp.	–	–	–	50.03 ± 0.50	69.85 ± 0.69	68.55 ± 0.22
YNA-C4	*Diaporthe* sp.	–	–	–	63.55 ± 0.01	74.87 ± 0.24	77.99 ± 0.01
GDA-1A2	*Fusarium* sp.	31.51 ± 1.25	11.11 ± 2.47	46.67 ± 0.22	58.26 ± 1.38	70.41 ± 2.89	72.33 ± 0.06
GDA-2A8	*Fusarium* sp.	45.21 ± 0.00	44.44 ± 0.82	43.33 ± 0.22	55.91 ± 1.93	63.71 ± 3.11	64.78 ± 0.22
GDA-2B7	*Fusarium* sp.	54.34 ± 1.67	48.15 ± 0.27	36.67 ± 0.22	–	–	–
GDA-2C9	*Fusarium* sp.	49.77 ± 0.42	33.33 ± 0.00	46.67 ± 0.89	–	–	–
GDA-2F9	*Fusarium* sp.	54.34 ± 0.42	48.15 ± 1.10	46.67 ± 0.89	–	–	–
GDA-3C4	*Fusarium* sp.	36.07 ± 1.67	22.22 ± 0.82	33.33 ± 0.89	55.32 ± 2.52	81.57 ± 0.58	72.33 ± 0.01
YNA-2C10	*Fusarium* sp.	54.34 ± 0.42	55.56 ± 0.00	56.67 ± 0.22	–	–	–
YNA-2C3	*Fusarium* sp.	81.74 ± 0.42	44.44 ± 0.82	36.67 ± 0.22	62.38 ± 0.13	56.45 ± 2.38	56.60 ± 1.23
YNA-2C6	*Fusarium* sp.	49.77 ± 0.42	62.96 ± 1.10	46.67 ± 1.56	–	–	–
GDA-1A7	*Lasiodiplodia* sp.	86.30 ± 1.25	59.26 ± 0.27	46.67 ± 0.22	74.13 ± 0.03	91.07 ± 0.02	79.25 ± 0.17
GDA-2A9	*Lasiodiplodia* sp.	63.47 ± 0.42	55.56 ± 0.00	60.00 ± 0.00	70.61 ± 0.03	89.39 ± 0.01	78.62 ± 0.13
GDA-2B1	*Lasiodiplodia* sp.	77.17 ± 0.42	48.15 ± 1.92	53.33 ± 1.56	72.96 ± 0.17	93.30 ± 0.00	73.58 ± 0.31
GDA-3C2	*Lasiodiplodia* sp.	49.77 ± 0.42	62.96 ± 0.27	50.00 ± 0.67	65.90 ± 0.30	93.30 ± 0.00	72.96 ± 0.06
YNA-1C2	*Lasiodiplodia* sp.	68.04 ± 0.42	70.37 ± 0.27	56.67 ± 0.89	75.31 ± 0.15	92.18 ± 0.01	75.47 ± 0.17
YNA-D3	*Lasiodiplodia* sp.	63.47 ± 0.42	74.07 ± 0.27	63.33 ± 0.89	75.90 ± 0.09	93.30 ± 0.00	76.73 ±0.10
YNA-1C3	*Neofusicoccum* sp.	49.77 ± 0.42	70.37 ± 0.27	46.67 ± 0.22	71.19 ± 0.13	92.18 ± 0.02	69.18 ± 0.15
YNA-A1	*Neopestalotiopsis* sp.	–	–	–	48.27 ± 1.69	64.82 ± 1.40	71.07 ± 0.06
GDA-2B6	*Nigrospora* sp.	40.64 ± 0.42	29.63 ± 0.27	40.00 ± 0.67	–	–	–
GDA-4C1	*Nigrospora* sp.	68.04 ± 0.42	62.96 ± 0.27	46.67 ± 3.56	64.73 ± 0.06	71.52 ± 1.70	71.70 ± 0.07
YNA-2C4	*Nigrospora* sp.	–	–	–	65.31 ± 1.38	79.34 ± 0.31	72.33 ± 0.03
YNA-A61	*Nigrospora* sp.	–	–	–	52.97 ± 0.01	78.78 ± 0.16	64.78 ± 0.10
YNA-A67	*Nigrospora* sp.	–	–	–	47.09 ± 0.08	93.30 ± 0.00	69.81 ± 0.00
YNA-A71	*Nigrospora* sp.	–	–	–	61.20 ± 0.02	68.73 ± 2.36	71.70 ± 0.02
GDA-5A7	*Paracamarosporium* sp.	–	–	–	50.62 ± 2.57	59.80 ± 1.40	71.70 ± 0.00
GDA-4C2	*Pseudopithomyces* sp.	36.07 ± 5.42	62.96 ± 0.27	33.33 ± 0.89	51.79 ± 1.50	65.38 ± 0.47	58.49 ± 1.45
YNA-1C1	*Trichoderma* sp.	45.21 ± 0.00	62.96 ± 0.27	60.00 ± 0.67	77.07 ± 0.02	92.74 ± 0.04	75.47 ± 0.17
GDA-1B3	*Trichosporon* sp.	–	–	–	51.21 ± 3.20	46.40 ± 1.74	74.21 ± 0.03
GDA-2B15	*Xylariaceae* sp.	–	–	–	54.14 ± 1.18	78.78 ± 0.16	72.33 ± 0.25

## Data Availability

Not applicable.

## Supplementary Materials

The following supporting information can be downloaded at: https://www.mdpi.com/article/10.3390/jof8111197/s1, Figure S1: Culture morphologies of 47 endophytic fungal strains obtained in this study (after 10 days on PDA).
