# Supplementary material for: Endophytic Fungi Associated with Aquilaria sinensis (Agarwood) from China Show Antagonism against Bacterial and Fungal Pathogens
Source: J Fungi (Basel). 2022 Nov 14;8(11):1197. doi: 10.3390/jof8111197 (PMC9697865; doi:10.3390/jof8111197)
Supplement: Supplementary file 1 [file jof-08-01197-s001.zip › jof-1988458-supplementary.pdf]

Supplementary

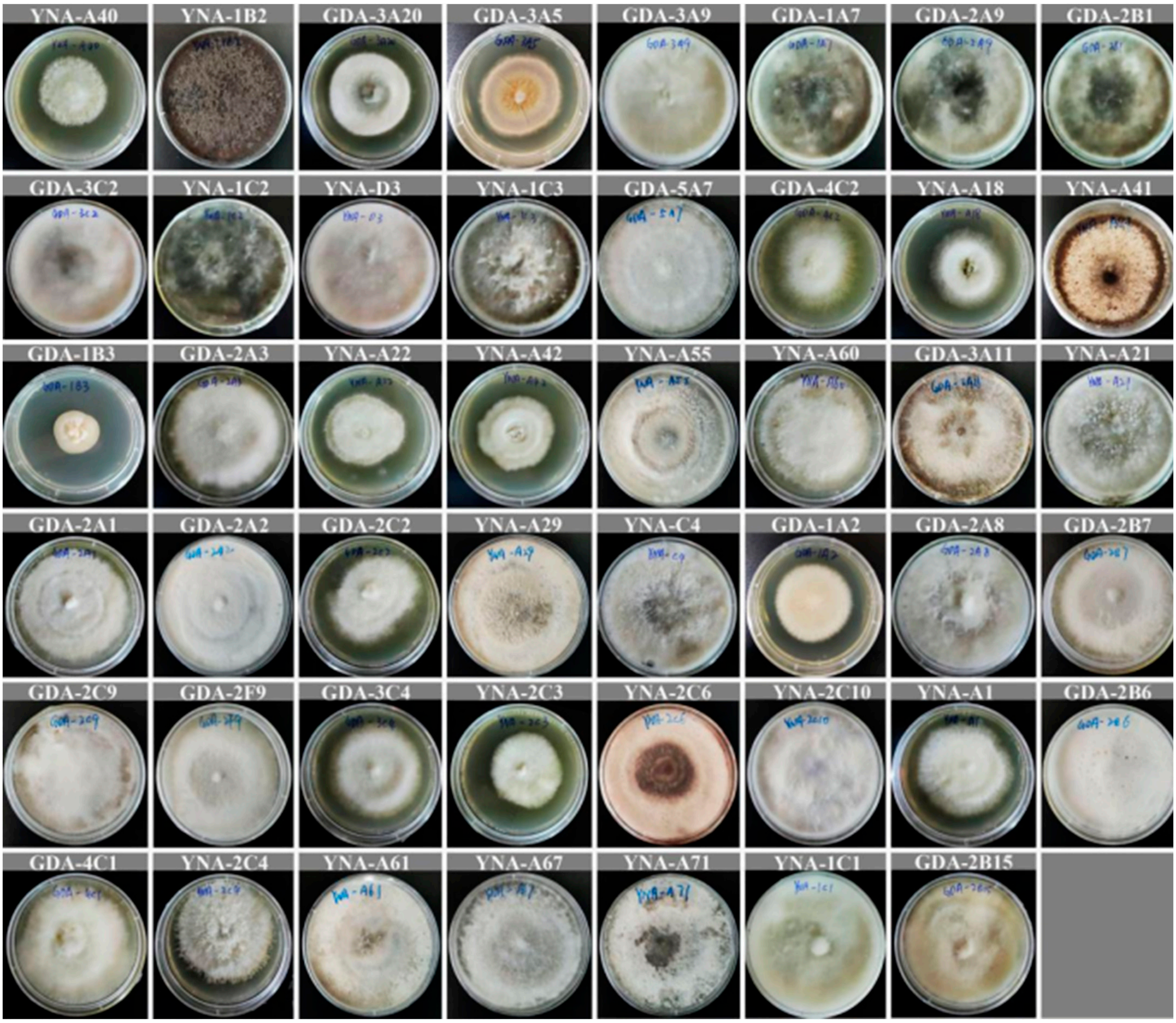

Figure S1. Culture morphologies of 47 endophytic fungal strains obtained in this study (after 10 days on PDA). They are arranged according to the order of strains as per Table 1.
